# Operation of Gastrotomy, Successfully Performed in a Case of Extra Uterine Pregnancy

**Published:** 1851-11

**Authors:** 


*Operation of Gastrotomy, successfully performed in a case of Extra Uterine Pregnancy.* By Doctors Bradley and Rogers, of Pineville, Monroe county, Alabama.

*Doctor Hester:* Dear Sir,—I send you, for publication, the following interesting case, the particulars of which were communicated to me by the gentlemen above named. There are several points connected with it, of great interest; and I would call attention especially to the large size of the child, and the extent of its connections with the parts of the mother. The gentlemen who had charge of the case deserve much credit, not less for skill in the treatment, by which, under very unfavorable circumstances, a favorable result was obtained, than for the operation itself.

Respectfully,

Wm. M. Boling.

The patient was a negro woman, twenty-eight years old, of medium size, the mother of seven children. Doctor Rogers saw her first on the 20th of June, 1849. She then seemed to be laboring under a very severe attack of colic, attended with constipation; she believed herself *pregnant, as she had not menstruated for six weeks.* The doctor treated her for similar attacks on the 29th of June, and on the 25th of August. Much difficulty was experienced each time in overcoming the constipation of the bowels. Again he saw her on the 1st of October, when she informed him that she had felt the motions of the child. The case now passed from under his charge, till the 10th February, 1850, when he was called, as was supposed, to deliver her. The patient thought herself in labor, and stated that she was in a great deal of pain. The os uteri was natural; she was not in labor. The breasts were flabby. She said that she had not felt the child move since the middle of November, 1849. She then had milk in her breasts. On the 15th of May, the doctor learned, through a member of the family, that she had menstruated several times, regularly as to periods, but profusely, and with relief.

On the 1st of February, 1851, Doctor Bradley saw her, with Doctor Rogers. The former gentleman gives the following statement of her condition at that time.

There was a large tumor filling the whole right lumbar region, extending above to the hypochondriac, and below so the iliac region, and somewhat to the left of the umbilicus. She was much emaciated, and had severe pains in the abdomen, extending also down her thighs. The tenderness of the abdomen was extreme. The tongue was coated slightly with a bluish fur, but was red at the edges. She had but little fever, however, the pulse being about natural in frequency, but small and feeble. She repeated, that when she was taken she had all the signs of pregnancy—cessation of catamenia, morning sickness, milk in the breasts, &c. She felt the motions of the child from the fourth or fifth month till the seventh or eighth, when she supposed it died.

Comparing the symptoms with the history of the case, we came to the conclusion, that it was a case of Extra Uterine Pregnancy.

The propriety of a surgical operation being suggested, the consent of patient and master were readily obtained. The proceeding was deferred till the 7th, with the view of preparing the patient.

The patient being placed in a suitable position, was, as a preparatory measure, brought fully under the influence of chloroform. Doctor Bradley operated, making an incision to the peritoneum, commencing half an inch below the umbilicus, extending down the linea alba to within an inch and a half of the pubes. The peritoneum was next elevated with forceps, and an incision made through it — a step which was attended with some difficulty, as the head of the fœtus was firmly attached to it. The incision was consequently extended two inches above the umbilicus.

We found the fœtœs in the right fallopian tube; it was fully formed, about the size of a seven months' child, and would have weighed about five pounds. No decomposition had taken place, except a little in the brain. It was firmly attached to the peritoneum anteriorly, posteriorly and laterally, and to the uterus below, so that the epidermis of the child would separate in passing the finger round to detach it. In consequence of the extent and firmness of the connection much time was consumed in the operation. The parts being carefully cleansed with a sponge, the edges of the wound were drawn together by four sutures, and adhesive strips, after which a bandage was applied round the abdomen. She was ordered an opiate.

The patient, on recovery from the anæsthetic stupor, had no knowledge of the performance of the operation. She was, however, sick at the stomach, and vomited several times. She had a comfortable night; moderate fever was developed, but by careful treatment was subdued. On the 11th the wound was dressed, and was found to be healing principally by the first intention. On the 16th she was regarded as convalescent, and in four weeks from the operation, she had entirely recovered. — *New Orleans Med. and Surg. Journal.*

